# Agreement between artificial intelligence and expert evaluation in assessing the quality of asthma health education videos on YouTube and Bilibili: cross-sectional study

**DOI:** 10.3389/fpubh.2026.1853895

**Published:** 2026-07-31

**Authors:** Kejun Li, Beilin Fu, Yinuo Chen, Ziteng Zhu, Daohui Wang, Xiangting Ge

**Affiliations:** 1 Department of Pulmonary and Critical Care Medicine, The Second Affiliated Hospital and Yuying Children’s Hospital of Wenzhou Medical University, Wenzhou, Zhejiang, China; 2 The Second Clinical Medical College, Wenzhou Medical University, Wenzhou, Zhejiang, China

**Keywords:** artificial intelligence, asthma, Global Quality Scale, health education video, mDISCERN, social media

## Abstract

**Background:**

Online video platforms such as YouTube and Bilibili have become important sources of health information for the public. However, the quality and reliability of online health education videos vary substantially. Artificial intelligence (AI), particularly large language models (LLMs), has recently shown potential for automated evaluation of health information, yet evidence regarding the agreement between AI-based and expert evaluations remains limited.

**Methods:**

A cross-sectional study was conducted to analyze asthma-related health education videos retrieved from YouTube and Bilibili. Two medical experts independently evaluated video quality using the Global Quality Scale (GQS) and modified DISCERN (mDISCERN). AI-assisted evaluation was performed using the GPT-4 large language model based on video transcripts under standardized prompts. Interrater agreement between experts was assessed using intraclass correlation coefficients (ICCs) and Spearman correlations. Agreement between AI and expert ratings was evaluated using Spearman correlation and Bland–Altman analysis. Multivariable linear regression was performed to identify factors associated with differences between AI and expert scores.

**Results:**

A total of 200 asthma-related health education videos were included, with 100 videos from each platform. AI ratings showed moderate correlations with expert ratings for both GQS (*ρ* = 0.55, *p* < 0.001) and mDISCERN (*ρ* = 0.49, *p* < 0.001). AI scores were slightly higher than expert scores, with mean differences of 0.24 for GQS and 0.60 for mDISCERN. Multivariable regression analysis identified platform (YouTube), source type (organizational source), and PEMAT actionability as significant factors associated with differences in mDISCERN scores, whereas no significant predictors were identified for GQS score differences.

**Conclusion:**

AI-generated evaluations demonstrated moderate positive correlations with expert assessments in evaluating the quality of asthma-related health education videos. AI may serve as a scalable tool for preliminary screening of online health information, although systematic differences remain across platforms and content characteristics. Expert evaluation remains essential to ensure the accuracy and reliability of medical information assessment.

## 1 Introduction

Asthma is one of the most common chronic respiratory diseases worldwide, affecting hundreds of millions of individuals and imposing a substantial burden on public health systems (1–3). Effective patient education is considered a critical component of long-term asthma management. By improving patients’ understanding of disease mechanisms, trigger factors, and evidence-based treatment strategies, educational interventions can significantly enhance disease control and overall quality of life (4–9). Therefore, providing accurate, understandable, and actionable health education information is essential for improving asthma management outcomes.

With the rapid development of digital media, online video platforms have become an increasingly important channel for the public to obtain health information. Platforms such as YouTube and Bilibili host a large number of health-related videos that can be widely accessed and disseminated (10–12). However, the quality and reliability of health information available on these platforms vary substantially (12, 13). Previous studies have reported that medical videos on social media may contain incomplete information, lack evidence-based recommendations, or even present misleading content (14). Consequently, evaluating the quality of online health education videos has become an important topic in digital health research.

Traditionally, the quality of health education videos has been assessed by medical experts using standardized evaluation instruments (15, 16). Commonly used tools include the Global Quality Scale (GQS), the modified DISCERN (mDISCERN) instrument, and the Patient Education Materials Assessment Tool (PEMAT). Although expert evaluation is generally regarded as the reference standard, this approach is time-consuming and difficult to apply to large-scale content screening across rapidly expanding online platforms.

Recent advances in artificial intelligence (AI), particularly LLMs, have demonstrated strong capabilities in natural language understanding and information analysis. These developments provide new opportunities for automated evaluation of online health information. Emerging evidence suggests that AI systems may be capable of assessing the quality of medical content, analyzing health-related texts, and supporting research in digital health communication (14, 17–19). Several studies have explored the use of AI to evaluate medical information on social media and have reported that AI-generated assessments may show a certain degree of agreement with expert judgments (20). However, empirical evidence regarding the agreement between AI-based evaluations and expert assessments remains limited, particularly in the context of health education videos across different online platforms.

In addition, characteristics of videos—such as source type, content structure, and presentation format—may influence both perceived information quality and the degree of agreement between AI and expert evaluations. Identifying factors associated with discrepancies between AI and expert ratings may help improve the reliability and interpretability of automated evaluation systems.

Therefore, this study analyzed asthma-related health education videos on YouTube and Bilibili with the following objectives: (1) to assess the agreement between artificial intelligence–generated ratings and expert evaluations in video quality assessment; (2) to compare rating patterns across different platforms; and (3) to explore whether video characteristics, including source, content type, and educational features, are associated with differences between AI and expert evaluations. The findings of this study may provide empirical evidence for the application of artificial intelligence in health information quality assessment and contribute to the development of human-AI collaborative evaluation frameworks in digital health communication.

## 2 Methods

### 2.1 Study design

This study was designed as a cross-sectional analysis aimed at evaluating the agreement between artificial intelligence–generated ratings and expert assessments in evaluating the quality of asthma-related health education videos. In addition, differences between platforms were examined, and potential factors associated with discrepancies between AI and expert ratings were explored.

### 2.2 Video selection

Asthma-related videos were retrieved from YouTube and Bilibili using the search keywords “asthma” and “哮喘.” The video retrieval and data extraction phase was executed from February 10 to February 25, 2026. To minimize personalized algorithmic bias, all searches were performed using a clean browser instance with cookies, cache, and localized historical data completely cleared, with results sorted exclusively by the platform-default ‘Relevance’ order.

Videos uploaded between December 31, 2015, and January 1, 2025, were eligible for screening. For each platform, the first 100 videos returned by the default relevance ranking were included for further evaluation. Videos were included if they met the following criteria: 1. The primary topic of the video was related to asthma; 2. The video was intended for public health education rather than professional medical training; 3. The video language matched the predefined study language.

Videos were excluded if they were duplicates, unrelated to asthma education, or lacked accessible video content or transcripts. Previous studies have suggested that analyzing the first 100 search results is sufficient to represent the majority of content encountered by users on video platforms (21). Therefore, only the first 100 eligible videos from each platform were included in the final dataset. The video selection process is shown in Supplementary Additional File 1.

### 2.3 Video characteristics

A standardized data extraction form was developed to collect baseline characteristics for each video. Extracted information included video duration, number of views, likes, comments, and other engagement metrics such as shares, favorites, and platform-specific interaction indicators.

In addition, videos were categorized according to source type, content category, and presentation format. Two reviewers independently classified all videos, and disagreements were resolved through discussion until consensus was reached.

#### 2.3.1 Video source

Videos were classified into four categories based on uploader identity: ① Medical source; ② Organizational source; ③ Individual source; ④ Commercial source.

#### 2.3.2 Video content

Videos were categorized according to their primary educational focus: ① Disease education; ② Diagnosis and treatment; ③ Daily management; ④ Prevention and first aid.

#### 2.3.3 Video format

Video presentation styles were categorized as: ① Solo narration; ② Question and answer; ③ Lecture or presentation (e.g., PPT or classroom style); ④ Animation or action-based explanation; ⑤ Medical scenario demonstration; ⑥ Television program or documentary; ⑦ Other formats.

### 2.4 Expert evaluation

Video quality and educational characteristics were independently assessed by two reviewers with medical training using three standardized evaluation instruments: the Global Quality Scale (GQS), the modified DISCERN (mDISCERN), and the Patient Education Materials Assessment Tool (PEMAT). The detailed scoring criteria for these evaluation tools are provided in Additional file 2–4 in the Supplementary materials.

The GQS and mDISCERN were used to evaluate the overall quality and reliability of the health information presented in the videos. The GQS is a 5-pointscale that provides a global assessment of video quality, flow, and usefulness for patients, while the mDISCERN instrument evaluates the reliability and completeness of medical information based on evidence-related criteria.

In addition, the Patient Education Materials Assessment Tool (PEMAT) was applied to assess the educational characteristics of the videos. Specifically, PEMAT evaluates two domains: understandability (PEMAT-U), which measures how easily consumers can understand the presented information, and actionability (PEMAT-A), which reflects whether the material provides clear actions that users can take based on the information. The two medical experts responsible for video evaluation possessed highly aligned professional backgrounds to ensure scoring objectivity. Both were certified male pulmonologists specializing in Pulmonary and Critical Care Medicine: Evaluator Wang holds a Master’s degree, and Evaluator Ge holds a Ph.D., with a mean of over 10 years of clinical and academic experience (aged 35–37). Both are native Chinese speakers with proficient professional English, enabling accurate, nuanced, and culturally sensitive interpretation of Bilibili (Chinese) and YouTube (English) health education content. A senior arbitrator—Doctor Xia, a chief physician with a doctoral degree and 30 years of clinical experience—was appointed to resolve any disagreements arising from the dual evaluation process.

Prior to formal evaluation, both reviewers received standardized training on all scoring instruments and jointly reviewed example videos to ensure consistent interpretation of the evaluation criteria. To eliminate target-audience selection bias, video audience classification was performed jointly by the two independent reviewers using a predefined operational protocol, with ambiguous or dual-purpose cases adjudicated by the senior third-party arbitrator. Each video was then independently rated by the two reviewers. Discrepancies between the two primary reviewers were resolved through a structured consensus protocol. For minor baseline variance (a score difference of 1 point), the two experts conducted face-to-face discussions to align their clinical and pedagogical focus. For larger discrepancies (≥2 points), a third senior respiratory consultant was consulted blindly to arbitrate and determine the final score.

Through this rigorous quality control pipeline, a 100% finalized consensus score was established for every video across all domains, which subsequently served as the gold-standard reference standard for the automated AI evaluation.

### 2.5 AI-assisted evaluation

All evaluations were performed using GPT-4 (gpt-4-turbo) between February 10 to February 25, 2026. To minimize variability in outputs, the temperature parameter was set to 0, top-p to 1.0, and both frequency and presence penalties to 0. This configuration reduces stochastic variation and improves response consistency. The complete prompt template is provided in Supplementary Additional File 5.

For each video, the complete spoken content was converted into text transcripts. When official subtitles or platform-generated captions were available, the corresponding subtitle text was directly extracted. For videos without available subtitles, an automatic speech recognition (ASR) system was used to generate transcripts from the audio track. To reduce potential transcription errors that could affect AI evaluation, a subset of transcripts was randomly selected and manually reviewed to confirm that no major semantic distortions were present.

The extracted transcript for each video was provided to the GPT-4 model together with a standardized evaluation prompt. The prompt instructed the GPT-4 to assess video quality based on the established criteria of the Global Quality Scale (GQS), modified DISCERN (mDISCERN), and the Patient Education Materials Assessment Tool (PEMAT). The model was explicitly instructed to follow the original scoring rules of each evaluation instrument and to return numerical ratings within the specified scoring ranges. This standardized prompt-based evaluation approach was designed to ensure that GPT-4-generated ratings adhered as closely as possible to the predefined scoring frameworks used in expert evaluation.

To minimize variability in GPT-4-generated outputs, all evaluations were conducted using identical model parameters. The temperature parameter was set to 0 and top-p was set to 1.0 to reduce stochastic variation and improve response consistency. Each video transcript was evaluated once under the same prompt and parameter configuration. By maintaining a fixed model version, prompt structure, and parameter settings, the GPT-4 evaluation process was standardized across all videos to enhance reproducibility.

### 2.6 Ethical considerations

This study analyzed publicly available online videos and did not involve human participants or identifiable personal data. Therefore, institutional review board approval and informed consent were not required.

### 2.7 Statistical analysis

Statistical analyses were performed using SPSS version 25.0 and R version 4.3.3. Continuous variables were presented as mean (SD) or median (IQR) depending on data distribution, while categorical variables were summarized as frequencies and percentages. Interrater agreement between the two expert reviewers for GQS and mDISCERN scores was assessed using intraclass correlation coefficients based on a two-way random-effects model [ICC (1, 2)]. Spearman rank correlation coefficients were calculated to evaluate rank consistency.

Agreement between AI-generated and expert ratings was evaluated using Spearman correlation analysis and Bland–Altman analysis. Mean differences and 95% limits of agreement were calculated. Comparisons between platforms were performed using the Mann–Whitney *U* test when variables were not normally distributed. To identify factors associated with discrepancies between GPT-4 and expert ratings, multivariable linear regression models were constructed. The dependent variable was the absolute difference between GPT-4 and expert scores. To achieve normal distribution and minimize skewness, highly skewed continuous independent variables (including views, likes, and durations) were log-transformed before being entered into the models. Independent variables included platform, video source, content category, presentation format, engagement metrics, and PEMAT scores. Multicollinearity was assessed using the variance inflation factor (VIF), with VIF < 3 considered acceptable. All statistical tests were two-sided, and *p* < 0.05 was considered statistically significant.

## 3 Results

### 3.1 Video characteristics

A total of 200 asthma-related health education videos were included in the analysis, comprising 100 videos from Bilibili and 100 from YouTube. The baseline characteristics of videos from Bilibili and YouTube are summarized in Supplementary Additional File 6. Overall, the quality of the included videos was moderate according to expert evaluations. The median expert Global Quality Scale (GQS) score was 3.0 (IQR 2.5–3.5) for Bilibili and 2.5 (IQR 2.0–3.5) for YouTube. Similarly, expert-rated modified DISCERN (mDISCERN) scores showed comparable distributions between platforms, with medians of 2.5 (IQR 2.0–3.0) for Bilibili and 2.0 (IQR 1.5–3.0) for YouTube.

**Figure 1:**
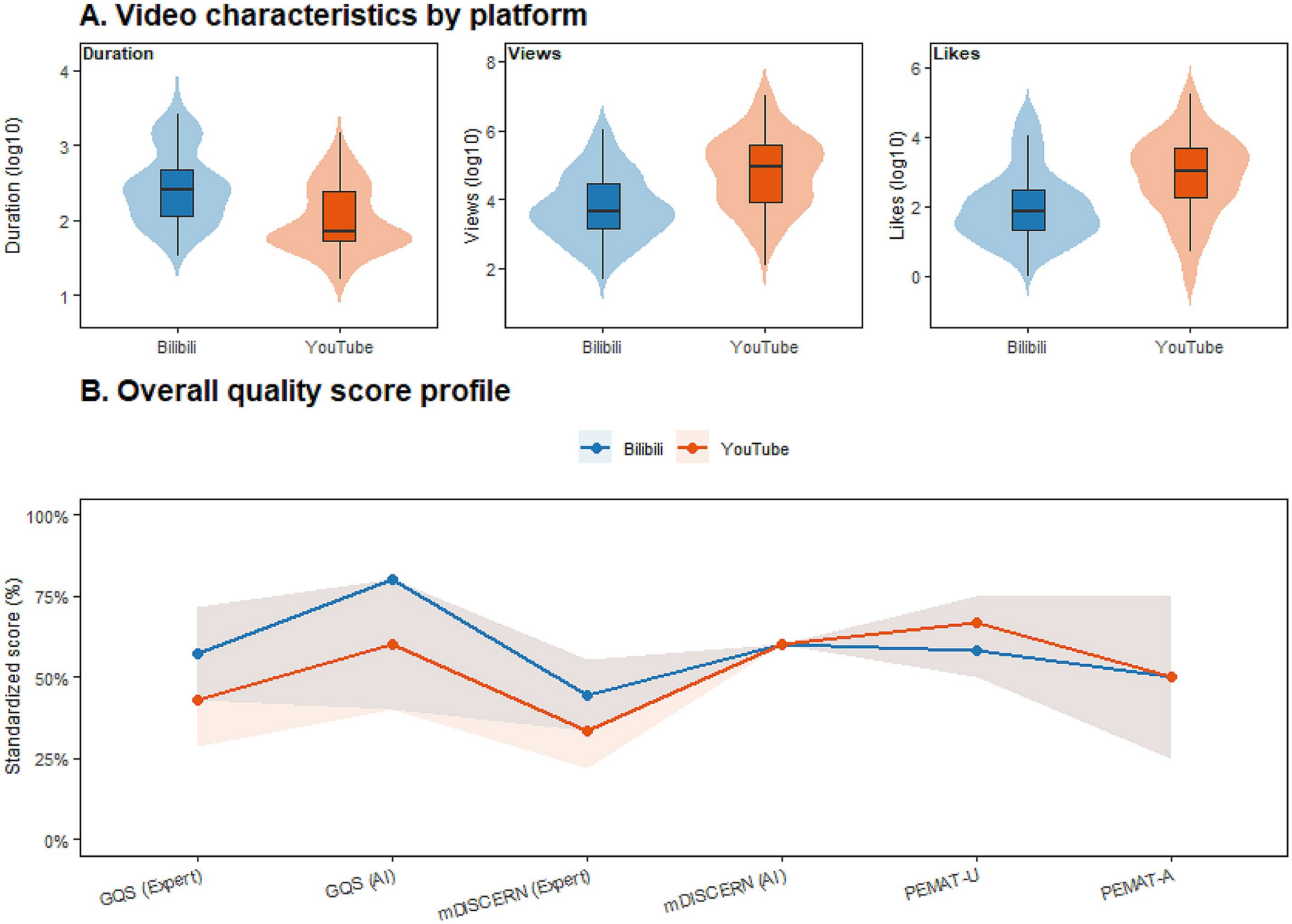
Video characteristics and overall quality score profiles by platform. **(A)** Distribution of video duration, views, and likes on Bilibili and YouTube. Violin plots represent the full distribution, with embedded boxplots indicating the median and interquartile range (IQR). All continuous variables were log_10_-transformed (log_10_ (*X* + 1)) to reduce skewness. **(B)** Overall quality score profiles across platforms. Median standardized scores (min–max normalization within each metric) are shown for expert-rated GQS, AI-rated GQS, expert-rated mDISCERN, AI-rated mDISCERN, PEMAT understandability (PEMAT-U), and PEMAT actionability (PEMAT-A). Shaded areas indicate the interquartile range (IQR).

Educational characteristics assessed using the Patient Education Materials Assessment Tool (PEMAT) were also similar across platforms. The median PEMAT understandability (PEMAT-U) score was 0.62 (IQR 0.54–0.77) for Bilibili and 0.69 (IQR 0.54–0.77) for YouTube, while the median PEMAT actionability (PEMAT-A) score was 0.50 (IQR 0.25–0.75) for both platforms. Overall distributions of video duration, view counts, and engagement metrics are presented in Figure 1.

### 3.2 Expert agreement

The initial interrater reliability and consistency between the two expert reviewers was low before consensus discussions. For GQS scores, the intraclass correlation coefficient (ICC) was 0.321, and the Spearman correlation coefficient was 0.365 (*p* < 0.001). For mDISCERN scores, the ICC was 0.338, and the Spearman correlation coefficient was 0.426 (*p* < 0.001). Specifically, across the evaluation of the 200 videos, perfect initial agreement (score difference = 0) was achieved in 69 videos for the GQS scale and 51 videos for the mDISCERN scale. Minor 1-point discrepancies that required face-to-face discussions occurred in 93 videos for mDISCERN and 79 videos for GQS. The third-party senior arbitrator was successfully triggered to resolve major discrepancies in 56 videos for mDISCERN and 52 videos for GQS. According to the standardized percentages, exact score agreement between reviewers occurred in 34.5% of videos for GQS and 25.5% for mDISCERN. When allowing a difference of ≤1 point, the agreement rates increased to 74.0% for GQS and 72.0% for mDISCERN (see Tables 1–3).

**Table 1:** Expert rating agreement.

Metric	ICC (2, 1)	Spearman *ρ*	*p* value	Exact agreement (%)	Agreement within 1 point (%)
GQS	0.321	0.365	<0.001	34.5	74
mDISCERN	0.338	0.426	<0.001	25.5	72

**Table 2:** AI vs. expert rating agreement.

Metric	Spearman *ρ*	*p* value	Mean difference (AI − expert)	95% limits of agreement
GQS	0.55	<0.001	0.24	−1.78 to 2.26
mDISCERN	0.49	<0.001	0.6	−1.25 to 2.45

**Table 3:** Platform-specific AI vs. expert agreement.

Platform	Metric	Spearman *ρ*	*p* value	Mean difference (AI − expert)
Bilibili	GQS	0.47	<0.001	0.28
Bilibili	mDISCERN	0.59	<0.001	0.55
YouTube	GQS	0.59	<0.001	0.2
YouTube	mDISCERN	0.41	<0.001	0.66

### 3.3 AI vs. expert agreement

Across the entire dataset, AI-generated ratings showed moderate correlations with expert evaluations for both scoring systems. For GQS, the Spearman correlation coefficient between AI and expert scores was *ρ* = 0.55 (*p* < 0.001). The mean difference (AI − expert) was 0.24, indicating that AI ratings were slightly higher on average. Bland–Altman analysis showed limits of agreement ranging from −1.78 to 2.26. For mDISCERN, the correlation between AI and expert ratings was *ρ* = 0.49 (*p* < 0.001). The mean difference was 0.60, again suggesting that AI tended to assign higher scores than experts. The limits of agreement ranged from −1.25 to 2.45.

**Figure 2:**
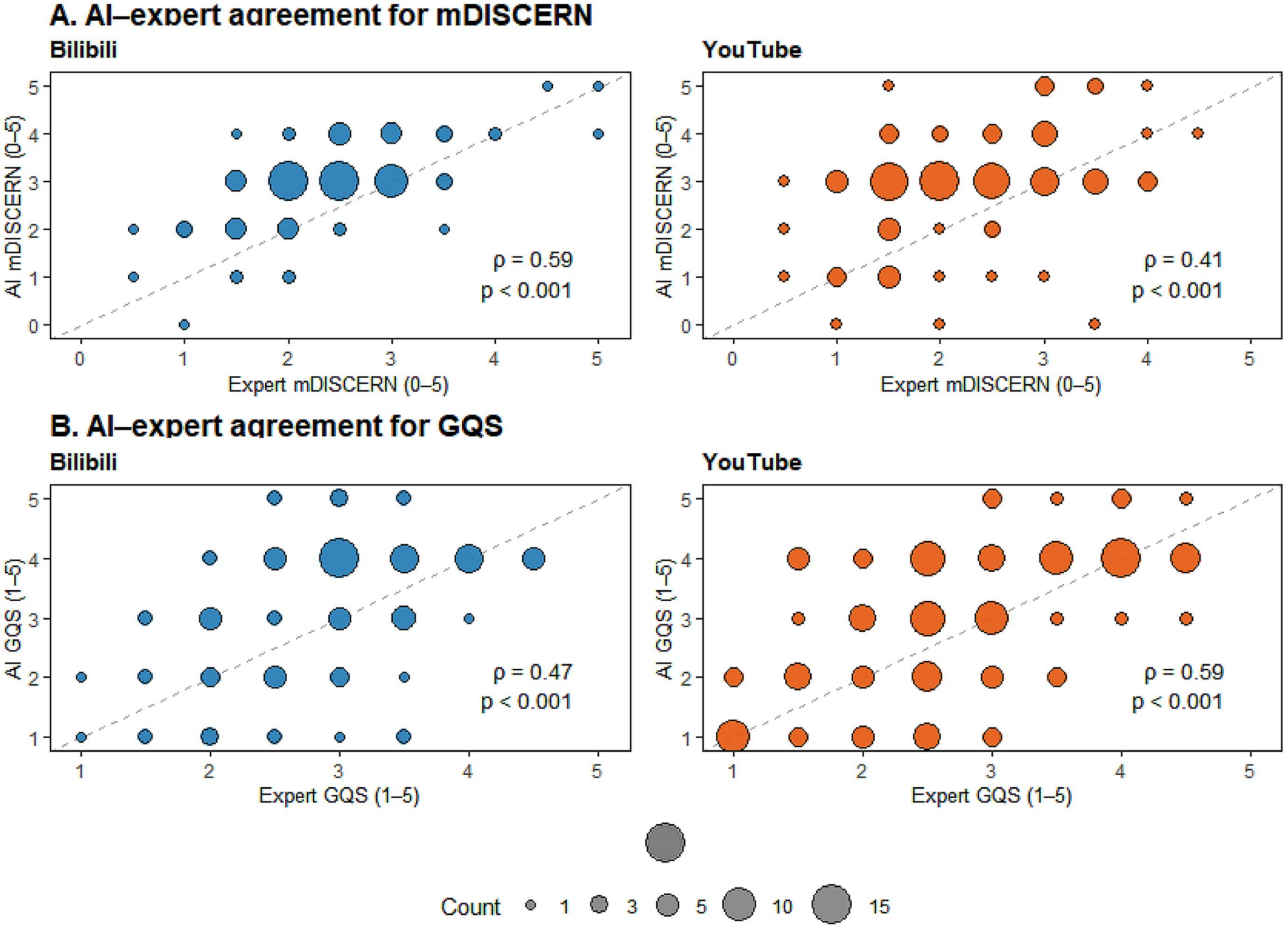
Agreement between artificial intelligence (AI) and expert evaluations for mDISCERN and Global Quality Scale (GQS) scores on Bilibili and YouTube. **(A)** Correlation between AI-generated and expert-assessed mDISCERN scores. **(B)** Correlation between AI-generated and expert-assessed GQS scores. Each panel presents platform-specific results for Bilibili (left) and YouTube (right). Bubble size represents the number of videos sharing the same paired score. The dashed diagonal line indicates perfect agreement (*y* = *x*). Spearman’s rank correlation coefficients (*ρ*) and corresponding *p* values are shown within each panel.

**Figure 3:**
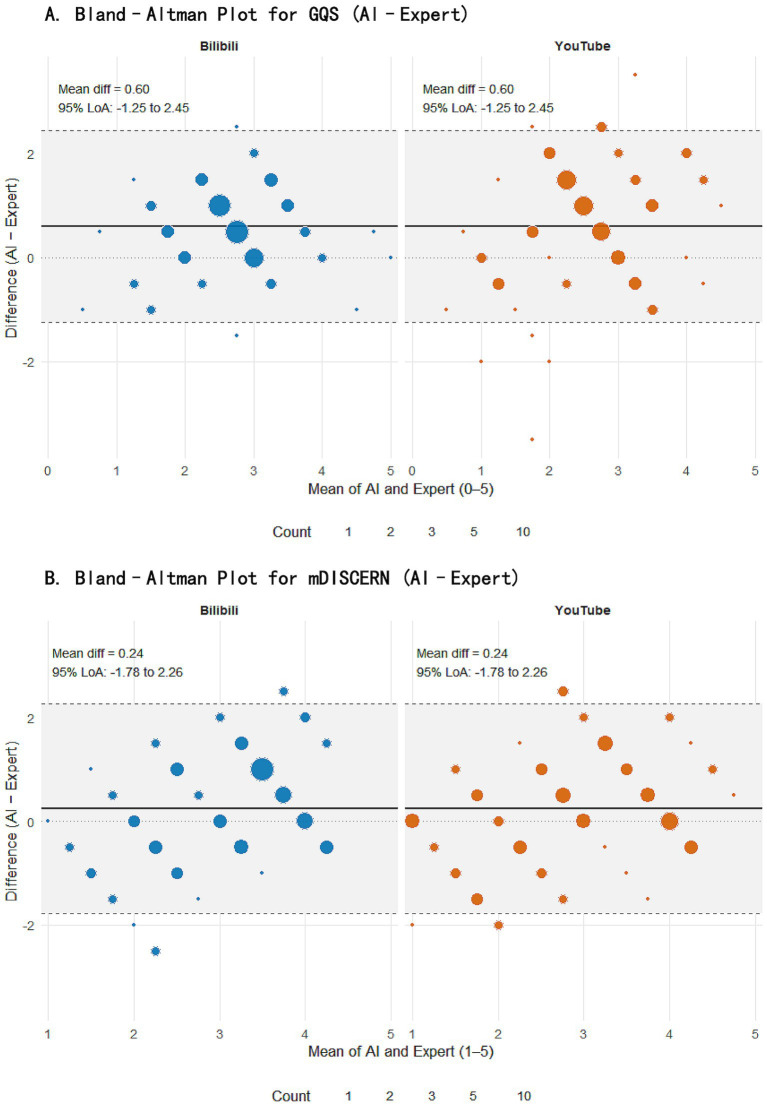
Agreement between AI-generated and expert-assessed scores evaluated using Bland–Altman plots. Panels display GQS **(A)** and mDISCERN **(B)** scores, stratified by platform (Bilibili and YouTube). The mean of AI and expert ratings is plotted against the signed difference (AI − Expert). Solid lines indicate mean bias, and dashed lines represent the 95% limits of agreement (mean ± 1.96 SD). Bubble size corresponds to the frequency of identical score pairs. Mean difference and limits of agreement were calculated using the pooled dataset.

Platform-specific analyses revealed moderate correlations on both platforms. For Bilibili, correlations were *ρ* = 0.47 for GQS and *ρ* = 0.59 for mDISCERN (both *p* < 0.001). For YouTube, correlations were *ρ* = 0.59 for GQS and *ρ* = 0.41 for mDISCERN (both *p* < 0.001). The distributions of paired AI and expert scores are illustrated in Figure 2, and the Bland–Altman agreement analyses are shown in Figure 3.

For mDISCERN, moderate positive correlations were observed on both Bilibili (*ρ* = 0.59, *p* < 0.001) and YouTube (*ρ* = 0.41, *p* < 0.001). For GQS, significant positive correlations were also identified on Bilibili (*ρ* = 0.47, *p* < 0.001) and YouTube (*ρ* = 0.59, *p* < 0.001).

### 3.4 Subgroup analysis

Differences in video quality according to video characteristics are summarized in Table 4. For video source, significant differences in expert-rated mDISCERN scores were observed across source categories on Bilibili (*p* = 0.015). On YouTube, video source was significantly associated with AI-rated GQS (*p* = 0.018), expert-rated GQS (*p =* 0.008), and expert-rated mDISCERN scores (*p* = 0.019). For video content, significant differences were observed in PEMAT actionability scores on Bilibili (*p* = 0.002), whereas no significant differences were found across content categories on YouTube. For video format, significant differences were identified in AI mDISCERN scores (*p* = 0.022) and expert mDISCERN scores (*p* = 0.026)on Bilibili, while no statistically significant differences were observed across format categories on YouTube.

**Table 4:** Subgroup analysis of AI and expert evaluation scores according to video characteristics on Bilibili and YouTube.

Platform	Category	Subgroup	AI GQS	AI mDISCERN	Expert GQS	Expert mDISCERN	PEMAT U	PEMAT A
Bilibili	Video source	Medical source (*n* = 49)	3.00 (1–5)	3.00 (1–5)	3.00 (1–4.5)	2.50 (1–5)	0.63 (0.08–0.92)	0.31 (0–1)
		Organizational source (*n* = 7)	4.00 (3–5)	3.00 (3–4)	3.25 (2–4.5)	2.75 (2–3.5)	0.67 (0.54–0.92)	0.50 (0.25–1)
		Commercial source (*n* = 2)	3.00 (2–4)	3.00 (3–3)	2.75 (2.5–3)	2.50 (2–3)	0.73 (0.69–0.77)	0.62 (0.25–1)
		Individual source (*n* = 42)	3.00 (1–5)	3.00 (0–4)	3.00(1.5–4.5)	2.00 (0.5–3.5)	0.54 (0.15–0.92)	0.38 (0–1)
		*p* value	0.346	0.114	0.518	0.015	0.377	0.584
	Video content	Disease education (*n* = 60)	3.50 (1–5)	3.00 (0–4)	3.00(1.5–4.5)	2.50 (1–5)	0.69 (0.23–0.92)	0.50 (0–1)
		Diagnosis and treatment (*n* = 26)	3.50 (1–5)	3.00 (1–5)	3.00 (1–4.5)	2.25 (1–5)	0.54 (0.08–0.77)	0.25 (0–1)
		Daily management (*n* = 9)	3.00 (1–5)	2.00 (1–3)	2.50 (1.5–4)	2.00 (0.5–3)	0.54 (0.15–0.92)	0.75 (0–1)
		Prevention & first aid (*n* = 5)	4.00 (3–5)	3.00 (3–4)	2.50 (2–3.5)	2.00 (1.5–3)	0.69 (0.31–0.85)	0.75 (0.50–1)
		*p* value	0.351	0.07	0.515	0.231	0.063	0.002
	Video format	Solo narration (*n* = 26)	3.00 (1–5)	3.00 (0–4)	3.00 (1–4)	2.00 (1–3.5)	0.54 (0.23–0.85)	0.50 (0–1)
		Question and Answer (*n* = 2)	3.50 (3–4)	3.50 (3–4)	3.00 (2–4)	2.50 (2–3)	0.46 (0.38–0.54)	0.62 (0.50–0.75)
		PPT or Class (*n* = 30)	4.00 (1–5)	3.00 (1–5)	3.00 (1–4.5)	2.50 (1–5)	0.62 (0.08–0.92)	0.25 (0–1)
		Animation/Action (*n* = 26)	4.00 (1–5)	3.00 (1–4)	3.00(1.5–4.5)	2.50 (1.5–3.5)	0.69 (0.31–0.92)	0.50 (0–1)
		Medical scenarios (*n* = 7)	4.00 (2–4)	3.00 (2–3)	3.00(1.5–3.5)	2.00 (0.5–3)	0.62 (0.46–0.85)	0.50 (0–1)
		TV show/documentary (*n* = 3)	4.00 (3–4)	3.00 (3–4)	3.00(1.5–4.5)	3.00 (3–3.5)	0.77 (0.54–0.85)	0.50 (0–0.75)
		Others (*n* = 6)	2.50 (1–3)	2.50 (1–3)	2.50 (2–3)	1.75 (0.5–3.5)	0.58 (0.15–0.77)	0.38 (0–1)
		*p* value	0.244	0.022	0.532	0.026	0.159	0.498
YouTube	Video source	Medical source (*n* = 45)	3.50 (1–5)	3.00 (1–5)	2.75(1.5–4.5)	2.50 (1–4)	0.69 (0.31–1)	0.50 (0–1)
		Organizational source (*n* = 19)	3.00 (1–5)	3.00 (0–5)	3.00 (1–4)	2.50 (0.5–3.5)	0.69 (0.38–0.85)	0.50 (0–1)
		Commercial source (*n* = 7)	2.00 (1–4)	3.00 (1–4)	2.50 (2–3)	1.50 (1.5–2.5)	0.62 (0.23–0.85)	0.25 (0.25–0.5)
		Individual source (*n* = 29)	2.00 (0–5)	3.00 (0–5)	2.00 (1–4.5)	1.50 (0.5–4.5)	0.62 (0.23–0.92)	0.50 (0–1)
		*p* value	0.018	0.133	0.008	0.019	0.562	0.063

	Video content	Disease education (*n* = 48)	3.00 (0–5)	3.00 (0–5)	2.50 (1–4.5)	2.00 (0.5–4.5)	0.62 (0.23–0.92)	0.50 (0–1)
		Diagnosis and treatment (*n* = 21)	3.00 (1–5)	3.00 (1–5)	2.50 (1–4)	2.00 (0.5–3.5)	0.62 (0.23–0.85)	0.50 (0–1)
		Daily management (*n* = 16)	3.00 (1–5)	3.00 (1–4)	2.50 (1–4)	2.00 (0.5–3.5)	0.69 (0.38–0.92)	0.50 (0–1)
		Prevention & first aid (*n* = 15)	3.00 (1–5)	3.00 (1–4)	2.50 (1–4)	2.00 (0.5–3.5)	0.69 (0.38–0.92)	0.50 (0–1)
		*p* value	0.391	0.858	0.509	0.741	0.571	0.978
	Video format	Solo narration (n = 31)	3.00 (0–5)	3.00 (0–5)	2.50 (1–4.5)	2.00 (0.5–4.5)	0.62 (0.23–0.92)	0.50 (0–1)
		Question and Answer (*n* = 3)	3.00 (2–4)	3.00 (2–4)	3.00 (2–4)	2.50 (1–3)	0.69 (0.38–0.85)	0.50 (0–0.75)
		PPT or Class (*n* = 20)	3.00 (1–5)	3.00 (1–5)	2.50 (1–4)	2.00 (0.5–3.5)	0.69 (0.38–0.92)	0.50 (0–1)
		Animation/Action (*n* = 18)	3.00 (1–5)	3.00 (1–5)	2.50 (1–4)	2.00 (0.5–3.5)	0.69 (0.38–0.92)	0.50 (0–1)
		Medical scenarios (*n* = 12)	3.00 (1–5)	3.00 (1–5)	2.50 (1–4)	2.00 (0.5–3.5)	0.69 (0.38–0.92)	0.50 (0–1)
		TV show/documentary (*n* = 9)	3.00 (1–5)	3.00 (1–5)	2.50 (1–4)	2.00 (0.5–3.5)	0.69 (0.38–0.92)	0.50 (0–1)
		Others (*n* = 7)	3.00 (1–5)	3.00 (1–5)	2.50 (1–4)	2.00 (0.5–3.5)	0.69 (0.38–0.92)	0.50 (0–1)
*p* value		0.318	0.742	0.441	0.692	0.587	0.821

Overall, the associations between video characteristics and quality indicators varied between platforms, with video source showing stronger associations on YouTube and video format demonstrating greater variability on Bilibili.

### 3.5 Regression analysis

Multivariable linear regression analyses were conducted to identify factors associated with differences between AI-generated and expert ratings. The dependent variable was the absolute difference between AI and expert scores. For the mDISCERN difference model, three variables were significantly associated with disagreement magnitude. Videos on YouTube showed larger AI–expert discrepancies than those on Bilibili (*β* = 0.319, *p* = 0.010). Videos from organizational sources were also associated with greater discrepancies compared with medical sources (*β* = 0.432, *p* = 0.008). In addition, higher PEMAT actionability scores were associated with smaller AI–expert differences (*β* = −0.478, *p* = 0.0196). No significant associations were identified between mDISCERN score differences and video content, presentation format, video duration, popularity indicators, or PEMAT understandability. For the GQS difference model, none of the examined variables were significantly associated with AI–expert rating differences.

**Figure 4:**
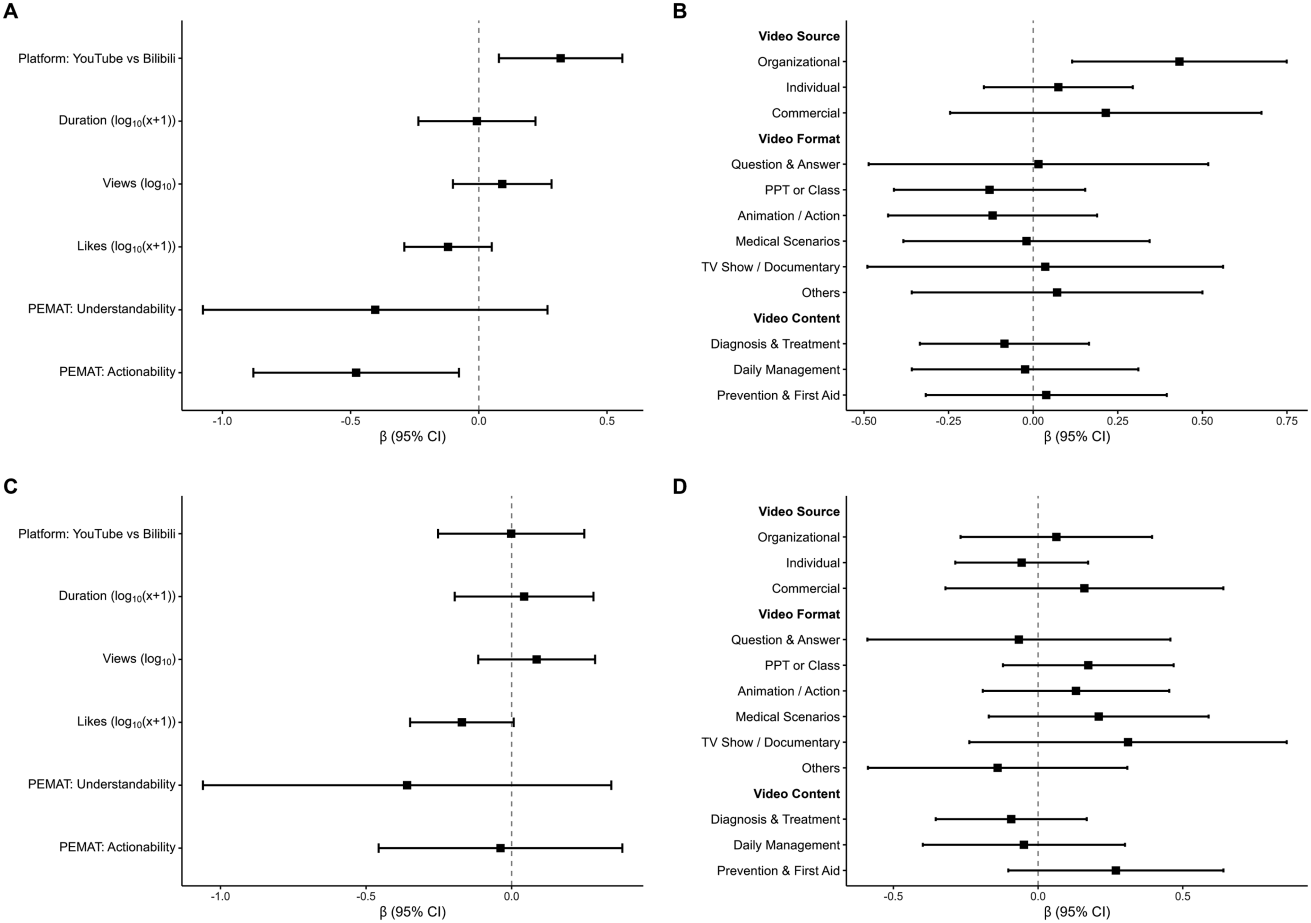
Multivariate linear regression analysis of factors associated with video quality scores. Forest plots displaying the multivariable linear regression analysis of video characteristics predicting discrepancy scores (calculated as AI score minus expert score). Panels **(A,B)** show predictors for the mDISCERN difference score; panels **(C,D)** show predictors for the Global Quality Scale (GQS) difference score. **(A,C)** (continuous & binary variables): variables of video duration and likes are log_10_(*x* + 1) transformed to stabilize variance and account for zero values. Views are presented as log_10_ transformed metadata. **(B,D)** (categorical variables): major categorical headers are bolded with individual sub-categories nested. Reference groups are medical source (video source), Solo narration (video format), and disease education (video content). Square symbols indicate the regression coefficients (*β*), and horizontal whiskers represent the 95% confidence intervals (CIs). The vertical dashed line indicates the null effect (*β* = 0).

Regression results are summarized in Figure 4.

## 4 Discussion

This study systematically evaluated the agreement between GPT-4-generated ratings and expert assessments of asthma-related health education videos on YouTube and Bilibili. GPT-4 ratings demonstrated moderate correlations with expert evaluations on both the Global Quality Scale (GQS) and modified DISCERN (mDISCERN), suggesting that large language models (LLMs) may hold utility for automated health information quality assessment. However, despite population-level alignment, systematic discrepancies persist at the individual video level, indicating that GPT-4 appraises information quality through an algorithmic framework distinct from that of clinical experts.

The low inter-rater reliability observed during the initial independent scoring phase (ICC: 0.321-0.338) is classified as ‘poor’ under strict clinical guidelines (22). However, this baseline variance closely aligns with previous multi-evaluator social media studies, where independent rater agreement typically ranges between 0.30 and 0.50 (23, 24). This variability is an inherent ‘field reality’ arising from the unstructured, multimodal nature of streaming videos, where presentation aesthetics confound subjective medical grading (25). Crucially, rather than undermining our findings, this prominent human variability underscores the scientific necessity for standardized, rule-based AI tools like GPT-4 to provide reproducible and scalable quality filtering for digital health content.

Consistent with prior work emphasizing that AI-based assessments cannot fully replicate expert judgment—particularly in domains necessitating specialized knowledge and evidence appraisal (19, 26)—GPT-4 ratings in our study were systematically higher than expert scores, with mean differences of 0.24 for GQS and 0.60 for mDISCERN. Bland–Altman analyses further revealed considerable individual-level disagreement between GPT-4 and expert ratings. This empirical gap suggests that while LLMs offer substantial efficiency gains for preliminary triage of large-scale online content, definitive quality assessments must continue to integrate human expertise to avert automated score inflation.

Furthermore, our multivariable regression analysis revealed that platform ecosystems, video sources, and content design significantly drive the scoring discrepancies between AI and human experts. Interestingly, YouTube demonstrated a much tighter human–AI consensus than Bilibili. This variance stems from their contrasting digital communication environments; YouTube functions as a mature, search-driven repository with a higher density of institutional creators producing structured, guideline-compliant contents (11, 27). Conversely, Bilibili operates as an engagement-heavy youth community (28) where creators routinely translate clinical facts into colloquial, narrative-driven ‘infotainment’ formats to optimize traffic (23). For instance, a video filled with emotional stories or friendly slang might easily trick a text-sensitive LLM into assigning an inflated rating due to its surface fluency (24), whereas medical experts would strictly penalize it for lacking scientific evidence (29). This pattern also explains why organizational video sources—which lean toward polished, standardized formatting—frequently trigger wider human–AI disagreements when explicit clinical guideline citations are absent (30–32).

Crucially, our findings revealed a compelling phenomenon regarding the PEMAT scores: while high video ‘understandability’ alone often biases the LLM toward overrated scores due to narrative fluency, high ‘actionability’ successfully bridges the human–AI evaluation gap. When a video provides clear, step-by-step guidance—such as demonstrating the exact sequential techniques of using a dry powder inhaler (‘First, open the cover; Second, breathe out entirely; Third, inhale deeply.’)—this precise, algorithmic phrasing removes semantic ambiguity for GPT-4. Simultaneously, explicit actionable directives leave less room for subjective interpretation among clinical experts, compelling both AI and human evaluators to anchor their criteria to the same objective behavioral metrics. Taken together, this systemic decoupling between online popularity (engagement metrics) and actual scientific rigor underscores the urgent public health necessity of deploying automated, rule-based screening frameworks alongside expert-in-the-loop validation for future digital health governance.

This study has several limitations. First, the AI evaluation in this study was exclusively transcript-based, omitting visual and non-textual multimedia features (such as presenter body language and graphical aids) that inherently influence human judgment. Future research should leverage multimodal medical AI models to bridge this textual-visual evaluation gap. The study included videos from only two platforms, YouTube and Bilibili. Although these platforms represent major sources of health information in international and Chinese contexts, the findings may not be generalizable to other social media platforms. The performance of LLMs may change over time as model architectures and training data evolve. Therefore, the results of this study may be partially dependent on the specific model version used. Additionally, this study restricted its AI-assisted evaluation to a single mainstream model version (GPT-4). While GPT-4 represents a peer-accepted benchmark for high-level textual coherence and structured evaluation, our cross-sectional approach did not conduct a horizontal comparison across multiple concurrent LLMs (e.g., Claude, Gemini, or domain-specific medical LLMs), which may exhibit varying performance profiles.

Future research should explore multimodal AI evaluation frameworks that integrate textual transcripts with visual features such as images, graphics, subtitles, and presentation structure. Incorporating multiple information modalities may allow AI systems to more accurately evaluate the educational quality of health videos (14, 18, 19, 33). To establish the generalizability of our findings, future research should investigate AI–expert alignment across a broader spectrum of disease topics, languages, and diverse social media platforms. Furthermore, conducting cross-model benchmarking—encompassing both open-source and proprietary LLMs—is essential to identify which architectures are optimally suited for specific medical domains. Ultimately, developing specialized medical AI frameworks or refined, domain-specific prompt engineering strategies will be critical to minimizing systematic rating variations and perfecting automated, scalable public health screening tools.

## 5 Conclusion

This study demonstrates that AI exhibits moderate rank consistency with expert evaluations in assessing asthma-related health education videos on YouTube and Bilibili, supporting its viability as a scalable instrument for preliminary screening of online health information. However, persistent systematic discrepancies—driven by platform-specific dynamics and content characteristics—reveal that current AI models lack the clinical acumen for independent quality control, and sole reliance on automated assessment risks misclassification of low-quality content. Accordingly, future efforts should adopt a “human-in-the-loop” framework (14), where in AI performs high-throughput triage and experts provide final validation, alongside refining AI training datasets with diverse cross-platform content to develop more robust digital health governance tools.

## Data Availability

The raw data supporting the conclusions of this article will be made available by the authors, without undue reservation.

## References

[1] Mims JW. Asthma: definitions and pathophysiology. Int Forum Allergy Rhinol. (2015) 5:S2–6. doi: 10.1002/ALR.21609, 26335832

[2] Gans MD Gavrilova T. Understanding the immunology of asthma: pathophysiology, biomarkers, and treatments for asthma endotypes. Paediatr Respir Rev. (2020) 36:118–27. doi: 10.1016/J.PRRV.2019.08.002, 31678040

[3] Holgate ST Wenzel S Postma DS Weiss ST Renz H Sly PD. Asthma. Nat Rev Dis Primers. (2015) 1:15025. doi: 10.1038/NRDP.2015.25, 27189668 PMC7096989

[4] Althoff MD Holguin F. Care of the patient with asthma. Ann Intern Med. (2025) 178:Itc81–itc96. doi: 10.7326/ANNALS-25-01034, 40489781

[5] Bateman ED Hurd SS Barnes PJ Bousquet J Drazen JM FitzGerald JM . Global strategy for asthma management and prevention: GINA executive summary. Eur Respir J. (2008) 31:143–78. doi: 10.1183/09031936.00138707, 18166595

[6] Strunk RC Mascia AV Lipkowitz MA Wolf SI. Rehabilitation of a patient with asthma in the outpatient setting. J Allergy Clin Immunol. (1991) 87:601–11. doi: 10.1016/0091-6749(91)90374-W, 2005311

[7] Celebi Sozener Z Aydin O Celik GE Mungan D. Effect of adherence on asthma control: more patient knowledge, more adherence, more control. Intern Med J. (2025) 55:1483–90. doi: 10.1111/IMJ.70135, 40590159

[8] Cai Q Jin M Li X Zhang J Xu Q Ye L . Effect of illness perceptions on asthma control and quality of life amongst adult outpatients with asthma in China. BMC Psychol. (2023) 11:68. doi: 10.1186/S40359-023-01097-3, 36907916 PMC10009986

[9] Koh YLE Chua KYK Ng DX Aau WK Tan NC. Assessing medication adherence in adults with asthma and its effect on rescue therapy for exacerbations. Front Pharmacol. (2025) 16:1516062. doi: 10.3389/FPHAR.2025.1516062, 40103590 PMC11914113

[10] Wang J Liu B Yang G Luo Y Lv N Shu X . Assessing the content and quality of GI bleeding information on Bilibili, TikTok, and YouTube: a cross-sectional study. Sci Rep. (2025) 15:14856. doi: 10.1038/S41598-025-98364-7, 40295710 PMC12038001

[11] Zhang Q Li Z Zhang H Han L Zhao S Jia S. YouTube and Bilibili as sources of information on oral cancer: cross-sectional content analysis study. Sci Rep. (2025) 15:21671. doi: 10.1038/S41598-025-02898-9, 40593960 PMC12214728

[12] Zhu Z Liu S Zhang R. Examining the persuasive effects of health communication in short videos: systematic review. J Med Internet Res. (2023) 25:e48508. doi: 10.2196/48508, 37831488 PMC10612001

[13] Dee EC Muralidhar V Butler SS Yu Z Sha ST Mahal BA . General and health-related internet use among Cancer survivors in the United States: a 2013-2018 cross-sectional analysis. J Natl Compr Cancer Netw. (2020) 18:1468–75. doi: 10.6004/JNCCN.2020.7591, 33152707

[14] Liu X Susarla A Padman R. Promoting health literacy with human-in-the-loop video understandability classification of YouTube videos: development and evaluation study. J Med Internet Res. (2025) 27:e56080. doi: 10.2196/56080, 40198918 PMC11984000

[15] Cui N Lu Y Cao Y Chen X Fu S Su Q. Quality assessment of TikTok as a source of information about mitral valve regurgitation in China: cross-sectional study. J Med Internet Res. (2024) 26:e55403. doi: 10.2196/55403, 39163110 PMC11372326

[16] Gong X Chen M Ning L Zeng L Dong B. The quality of short videos as a source of coronary heart disease information on TikTok: cross-sectional study. JMIR Form Res. (2024) 8:e51513. doi: 10.2196/51513, 39226540 PMC11408897

[17] Rodriguez-Rodriguez AM De la Fuente-Costa M Escalera-de la Riva M Perez-Dominguez B Paseiro-Ares G Casaña J . AI-enhanced evaluation of YouTube content on post-surgical incontinence following pelvic cancer treatment. SSM Popul Health. (2024) 26:101677. doi: 10.1016/J.SSMPH.2024.101677, 38766549 PMC11101902

[18] Kou T. Liu X. Zhang Z. Li C. Wu H. Min X. . Subjective-aligned dataset and metric for text-to-video quality assessment Proceedings of the 32nd ACM International Conference on Multimedia: 2024 (2024) 7793–7802

[19] Arcot D Pondicherry N Chakraborty S. Evaluating the quality of health information: comparison of human and artificial intelligence. Neurogastroenterol Motil. (2025) 38:e70164. doi: 10.1111/NMO.70164, 40993963 PMC13244121

[20] Chongqing Popular Science Research Association CSoLM Chinese Society of Toxicology Geriatric Disease Prevention and Control Professional Committee of Chinese Preventive Medicine Association Chongqing Medical Association Luo Y Wang CX. Chinese expert consensus on the framework of life-cycle active health popular science. Lab Med Clin. (2025) 22:145–50. doi: 10.3969/j.issn.1672-9455.2025.02.001 (In Chinese)

[21] Gonzalez-Estrada A Cuervo-Pardo L Ghosh B Smith M Pazheri F Zell K . Popular on YouTube: a critical appraisal of the educational quality of information regarding asthma. Allergy Asthma Proc. (2015) 36:e121–6. doi: 10.2500/AAP.2015.36.3890, 26534743

[22] Koo TK Li MY. A guideline of selecting and reporting Intraclass correlation coefficients for reliability research. J Chiropr Med. (2016) 15:155–63. doi: 10.1016/J.JCM.2016.02.012, 27330520 PMC4913118

[23] Xu J He Q Chen J Peng X Liang J. A cross-cultural comparison of educational video quality on college students' anxiety and depression: a cross-sectional content analysis of YouTube and Bilibili. Front Public Health. (2026) 14:1783552. doi: 10.3389/FPUBH.2026.1783552, 41889626 PMC13013497

[24] Qiu X Zhou Y Yuan T Wang Q Zou Z Wang L . A cross-sectional analysis of the quality and characteristics of sleep apnea hypopnea syndrome videos on YouTube, Bilibili, and TikTok. Sci Rep. (2026) 16:4070. doi: 10.1038/S41598-025-34182-1, 41526588 PMC12855175

[25] Kusama H Takahashi Y Orihara S Adachi K Ishizuka Y Semba R . Assessing the reliability and validity of principles for health-related information on social media (PRHISM) for evaluating breast Cancer treatment videos on YouTube: instrument validation study. JMIR Infodemiol. (2025) 5:e66416. doi: 10.2196/66416, 40498658 PMC12175871

[26] Khalil M Mohamed F Shoufan A. Evaluating the quality of medical content on YouTube using large language models. Sci Rep. (2025) 15:9906. doi: 10.1038/S41598-025-94208-6, 40121315 PMC11929840

[27] Lian D Zhang Y Liu Z Lin Y Ai M Chen Y . Public health implications of allergic rhinitis information on YouTube and Bilibili: a cross-cultural analysis of content quality, engagement, and seasonal trends. BMC Public Health. (2026). 26:28015. doi: 10.1186/S12889-026-28015-7, 42271340 PMC13479579

[28] Dzau V DeStefano L. Supporting access to reliable health information with public–private collaboration. N Engl J Med. (2026) 395:212–4. doi: 10.1056/NEJMP2602837, 42269148

[29] Burstin H Curry S Ranney ML Arora V Wachler BB Chou WS . Identifying credible sources of health information in social media: phase 2-considerations for non-accredited nonprofit organizations, for-profit entities, and individual sources. NAM Perspect. (2023) 2023:202305b. doi: 10.31478/202305B, 37916063 PMC10617996

[30] Xuan W Tian K Hao L. Quality assessment of short videos on health science popularization in China: scale development and validation. Front Public Health. (2025) 13:1640105. doi: 10.3389/FPUBH.2025.1640105, 41048298 PMC12488633

[31] Yelbay M Karacaoglu F Kurumlu M. Evaluation of the quality and reliability of Turkish and English YouTube video contents on gingival recession treatment. Sci Rep. (2025) 15:14444. doi: 10.1038/S41598-025-99371-4, 40281082 PMC12032028

[32] Yang G Li X Duan X. Assessment of health information in Chinese atopic dermatitis-related videos: a cross-sectional study. Digit Health. (2025) 11:20552076251346579. doi: 10.1177/20552076251346579, 40463986 PMC12130654

[33] Zhou S Huang M Wei J Fang H Yang W Yu H . Benchmark evaluation of video large language models in quality assessment of science popularization videos for dry eye. Sci Rep. (2026) 16:8756. doi: 10.1038/s41598-026-39444-0 PMC12982513 41688579

