# Supplementary material for: Agreement between artificial intelligence and expert evaluation in assessing the quality of asthma health education videos on YouTube and Bilibili: cross-sectional study
Source: Front Public Health. 2026 Jul 31;14:1853895. doi: 10.3389/fpubh.2026.1853895 (PMC13473413; doi:10.3389/fpubh.2026.1853895)
Supplement: Supplementary file 1 [file Data_Sheet_1.DOCX]

# Supplementary Materials

Additional file 1. Flowchart of video selection.

Additional file 2. Global Quality Scale (GQS) scoring criteria.

Additional file 3. Modified DISCERN (mDISCERN) evaluation items.

Additional file 4. PEMAT evaluation criteria for audiovisual materials.

Additional file 5. Prompt used for AI-assisted video quality evaluation.

Additional file 6. Baseline characteristics of videos from Bilibili and YouTube.

## Additional file 1. Flowchart of Video Inclusion and Exclusion

Flowchart illustrating the process of video identification, screening, eligibility assessment, and final inclusion of asthma-related health education videos.

## Additional file 2. Global Quality Scale (GQS) for Video Quality Evaluation

| **Score** | **Quality Level** | **Description** |
| --- | --- | --- |
| 1 | Poor quality | Poor quality and flow; most information is missing or inaccurate; not useful for patients. |
| 2 | Generally poor | Limited information provided; important topics are missing; low usefulness for patient education. |
| 3 | Moderate quality | Some relevant information is provided but incomplete; moderate usefulness for patients. |
| 4 | Good quality | Most relevant information is included; clear structure and useful for patient education. |
| 5 | Excellent quality | Comprehensive, well-structured, and highly useful for patient education. |

## Additional file 3. Modified DISCERN (mDISCERN) Scale for Asthma Information Quality

| **Item** | **Description** |
| --- | --- |
| Item 1 | Are the aims of the video clear and concise? |
| Item 2 | Are reliable sources of information cited? |
| Item 3 | Is the information presented balanced and unbiased? |
| Item 4 | Are additional sources of information provided for further reference? |
| Item 5 | Is the information presented accurate and non-controversial? |

## Additional file 4. PEMAT Evaluation Criteria for Asthma Audiovisual Materials

| **Dimension** | **Category** | **Specific Item** |
| --- | --- | --- |
| Understandability | Content | 1. The purpose of the audiovisual material is clearly stated. |
|  | Word choice and style | 2. Common, everyday language is used. |
|  |  | 3. Medical terminology is explained when used. |
|  |  | 4. Active voice is used. |
|  | Organization | 5. Information is presented in short, manageable sections. |
|  |  | 6. Sections include clear headings (mark N/A if the material is very brief). |
|  |  | 7. Information is presented in logical order. |
|  |  | 8. A summary of key points is provided (mark N/A if the material is very brief). |
|  | Layout and design | 9. Visual cues highlight key information. |
|  |  | 10. On-screen text is easy to read (mark N/A if no text is present). |
|  |  | 11. Audio is clear and easy to understand (mark N/A if no narration is present). |
|  | Visual aids | 12. Illustrations or photographs are clear and simple (mark N/A if none are used). |
|  |  | 13. Tables (if present) include clear and understandable headings. |
| Actionability | Action guidance | 14. The material clearly identifies at least one action the user can take. |
|  |  | 15. Actions are described in a way that directly addresses the user. |
|  |  | 16. Actions are broken down into specific steps. |
|  |  | 17. Explanations are provided on how to perform actions using visual aids (mark N/A if no visual aids are present). |

**Scoring method**Agree = 2 points；Disagree = 1 point； Not applicable (N/A) = 0 points

## Additional file 5. Prompt Used for AI-Assisted Video Quality Evaluation

| **Section** | **Prompt content** |
| --- | --- |
| System instruction | You are a medical information evaluator with expertise in assessing the quality and reliability of health education materials. |
| Task description | Please evaluate the following transcript of a health education video related to asthma. Your evaluation should follow the scoring criteria of the Global Quality Scale (GQS) and modified DISCERN (mDISCERN). |
| Evaluation instructions | Based on the transcript, assign numerical scores according to the definitions of each scale. Do not provide explanations; only output the numerical scores. |
|  | **Global Quality Scale (GQS):** score from 1 to 5, where higher scores indicate better overall quality and usefulness of the health information. |
| Scoring scales | **Modified DISCERN (mDISCERN):** score from 0 to 5 based on the reliability and quality of information provided in the video. |
| Output format | Please return the results in the following format: |
|  | GQS: X |
|  | mDISCERN: X |
| Input text | Transcript of the video: [video transcript inserted here] |

## Model version: We used the GPT-4 API (gpt-4-turbo). All evaluations were performed between February 10 to February 25, 2026，using the identical model checkpoint to avoid cross-version variability.

## Parameter settings: To ensure reproducibility and minimize stochastic variation, we set the temperature parameter to 0 and top-p to 1.0. Frequency penalty and presence penalty were both set to 0. These settings were chosen because our task—standardized quality scoring of health education videos—requires deterministic, rule-based outputs rather than creative or diverse responses. A temperature of 0 forces the model to select the most probable next token at each step, substantially reducing output randomness and enhancing consistency across repeated evaluations.

## Complete prompt: The standardized prompt template used for GPT-4 evaluation has been provided in Supplementary Additional file 5. The prompt includes role-setting instructions, explicit scoring criteria for GQS, mDISCERN, and PEMAT, and structured output format requirements.

## Additional file 6. Basic Characteristics of Videos on Bilibili and YouTube

| Variables | Bilibili (n=100), median (min, max) | YouTube (n=100), median (min, max) | Total (n=200), median (min, max) | Mann-Whitney test | |
| --- | --- | --- | --- | --- | --- |
|  |  |  |  | Z score | P value |
| duration (seconds) | 248.50 (32.00, 5018.00) | 69.00 (15.00, 1505.00) | 147.50 (15.00, 5018.00) | 5.914 | <0.001 |
| Likes | 70.00 (0.00, 61000.00) | 1054.00 (0.00, 170000.00) | 232.00 (0.00, 170000.00) | -5.573 | <0.001 |
| Comments | 7.00 (0.00, 3140.00) | 32.00 (0.00, 19890.00) | 13.00 (0.00, 19890.00) | -1.629 | 0.102 |
| Views | 4482.50 (46.00, 1093000.00) | 87000.00 (123.00, 10800000.00) | 12500.00 (46.00, 10800000.00) | -6.377 | <0.001 |
| Expert GQS mean | 3.00 (1.00, 4.50) | 2.50 (1.00, 4.50) | 3.00 (1.00, 4.50) | 1.682 | 0.088 |
| AI GQS mean | 3.50 (1.00, 5.00) | 3.50 (1.00, 5.00) | 3.50 (1.00, 5.00) | 3.50 (1.00, 5.00) | 3.50 (1.00, 5.00) |
| Expert DISCERN mean | 2.50 (0.50, 5.00) | 2.00 (0.50, 4.50) | 2.50 (0.50, 5.00) | 1.274 | 0.196 |
| AI DISCERN mean | 3.00 (0.00, 5.00) | 3.00 (0.00, 5.00) | 3.00 (0.00, 5.00) | 3.00 (0.00, 5.00) | 3.00 (0.00, 5.00) |
| PEMAT Understandability | 0.62 (0.08, 0.92) | 0.69 (0.23, 1.00) | 0.62 (0.08, 1.00) | -1.745 | 0.078 |
| PEMAT Actionability | 0.50 (0.00, 1.00) | 0.50 (0.00, 1.00) | 0.50 (0.00, 1.00) | -1.889 | 0.052 |
